# Catalyzing computational biology research at an academic institute through an interest network

**DOI:** 10.1371/journal.pcbi.1013453

**Published:** 2025-09-10

**Authors:** Jaroslav Zak, Ian Newman, Daniel J. Montiel Garcia, Daniele Parisi, Janet Joy, Steven R. Head, Jean-Christophe Ducom, Padmaja Natarajan, Haissi Cui, Sabah Ul-Hasan

**Affiliations:** 1 Department of Immunology and Microbiology, The Scripps Research Institute, La Jolla, California, United States of America; 2 Huntsman Cancer Institute, University of Utah School of Medicine, Salt Lake City, Utah, United States of America; 3 Department of Radiation Oncology, University of Utah School of Medicine, Salt Lake City, Utah, United States of America; 4 Department of Integrative Structural and Computational Biology, The Scripps Research Institute, La Jolla, California, United States of America; 5 Genomics Core, The Scripps Research Institute, La Jolla, California, United States of America; 6 Information Technology Services, The Scripps Research Institute, La Jolla, California, United States of America; 7 Center for Computational Biology and Bioinformatics, The Scripps Research Institute, La Jolla, California, United States of America; 8 Department of Chemistry, University of Toronto, Toronto, Ontario, Canada; 9 Independent Researcher, Albuquerque, New Mexico, United States of America; Montreal, CANADA

## Abstract

Biology has been transformed by the rapid development of computing and the concurrent rise of data-rich approaches such as, omics or high-resolution imaging. However, there is a persistent computational skills gap in the biomedical research workforce. Inherent limitations of classroom teaching and institutional core support highlight the need for accessible ways for researchers to explore developments in computational biology. An analysis of the Scripps Research Genomics Core revealed increases in the total number and diversity of experiments: the share of experiments other than bulk RNA- or DNA-sequencing increased from 34% to 60% within 10 years, requiring more tailored computational analyses. These challenges were tackled by forming a volunteer-led affinity group of approximately 300 academic biomedical researchers interested in computational biology, referred to as the Computational Biology and Bioinformatics (CBB) affinity group. This adaptive group has provided continuing education and networking opportunities through seminars, workshops, and coding sessions while evolving along with the needs of its members. A survey of CBB’s impact confirmed the group’s events increased the members’ exposure to computational biology educational and research events (79% respondents) and networking opportunities (61% respondents). Thus, volunteer-led affinity groups may be a viable complement to traditional institutional resources for enhancing the application of computing in biomedical research.

## Author summary

Scientists have widely adopted computing in their research, and the relative importance of computational methods in biology continues to increase. However, a persistent gap exists between the actual and required skill levels for utilization of latest computational methods, driven in part by inherent limitations of formal teaching and institutional resources. In this work, we examined the computational research activity at Scripps Research, an independent academic biomedical research institute, and found an increase in the total number and diversity of experiments requiring computational analyses. We also found that the majority of laboratories, including those not specialized in computational research, utilize the institute’s high-performance computing resources, highlighting the widespread adoption of computing across multiple areas of biology. We describe the formation of a volunteer-led informal affinity group of scientists interested in computational biology and its activities, including seminars, workshops, and coding sessions. A survey indicated that the group has increased the members’ exposure to computational biology and networking opportunities, suggesting its utility in complementing existing institutional resources.

## Introduction

Since the mid-1960s [1], biology has gradually transformed into an information science. Over this period, the volume of biomedical literature has increased exponentially and has been accompanied by an explosion of -omics data. Both the volume of scientific publication [2] and -omics data [3] are increasing exponentially. For example, public databases of major types of genomic and proteomic data, such as dbSNP, EGA, and ENA, have experienced double-digit percent annual growth in the number of records and sequences [4]. Similarly, the number of tools published to analyze -omics data has experienced faster than linear growth [5]. Deep learning based approaches have resulted in paradigm shifts in the area of protein prediction, raising expectations for their transformative effects in other areas of biology, such as drug discovery [6].

As interaction with large biomedical data has become ubiquitous across research disciplines, so too has the rate of interaction between computational tools and researchers who would otherwise not describe themselves as computational biologists [7]. Without consistent efforts to stay informed, they risk using outdated methods or misapplying tools, resulting in inefficiencies and errors. For instance, the authors of TopHat noted in 2017 that TopHat continues to be used for RNA-seq read alignment more each year, nearly 10 years after its publication, while superior versions have long been available (first TopHat2, then HISAT, then HISAT2) [8]. Similarly, default settings in software such as Microsoft Excel have led to preventable errors, such as converting gene names into calendar dates, causing data loss [9,10]. These issues underscore the need for both awareness of new tools and appropriate training to use them effectively.

Traditionally, in academic settings, a large part of the dissemination and training for new techniques happens amongst colleagues within the same laboratory group or department. This mechanism can be less effective in multidisciplinary groups [11], where computational tools may not be the central focus. Institutional strategies for alleviating this challenge have included outsourcing computational analyses to computational biology cores, encouraging collaboration outside of the department (e.g., with computational biology laboratory groups), and offering formal training courses. Yet, significant disparities in computational literacy and utilization persist [12–14].

There is a long-standing gap between the expected and actual levels of computational literacy in the research workforce [12,13]. Tan and colleagues proposed a minimum set of skills for university graduates to meet the informatics needs of the “-omics era,” including the command of bioinformatics tools and a basic understanding of programming languages [15]. A survey of 1260 faculty by the NSF Network for Integrating Bioinformatics into Life Sciences Education defined core competencies for undergraduate students in the life sciences, which include accessing genomic data, genomic tools, some experience with command line tools, and writing simple scripts [16]. However, these core competencies are not universally held by researchers as the divide between “dry lab” and “wet lab” biologists persists. A recurring recommendation is for the education curricula to be updated to match the demands of contemporary biology [17], but formal education alone may not be able to keep pace with rapidly evolving computational technologies, nor can it be expected to deliver the tailored information needed for individual research projects. Further, as technological advances continue beyond the completion of formal undergraduate and graduate classes, there is a need for efficient ways of updating computational knowledge and skills for graduated practitioners. Although empirical data on the skills gap in the biomedical research job market is lacking, data from other fields suggest evolving methods create demand for the latest computational skills, such as in data science [18,19].

The adoption of computational tools in research often mirrors broader trends in technology adoption, leading to near-universal uptake in some areas. For example, the National Institutes of Health no longer accept completing grant applications on paper [20]. Electronic slides prepared in presentation software have become the universal format of scientific conferences, and electronic classroom tools have also reached near universal adoption [21]. Similar trends are observed in other sectors, for example, electronic health records are gradually replacing paper copies nationwide [22]. Recent advances in artificial intelligence (AI), including interactive chatbots such as ChatGPT, suggest that AI-powered tools could be headed towards near-universal adoption in the long term. Indeed, in the 3 months following the public release of ChatGPT, an estimated 10% of scientific papers were completed with the assistance of ChatGPT or similar large language model tool, a striking statistic given that many papers published during this time were peer reviewed before ChatGPT was publicly released [23]. A 2024 survey of 3,839 students in 16 countries found that over 86% of students use AI tools in some form [24], and the estimated usage was even higher in a 2025 survey of students in which 92% reported AI use and a reported increase in generative AI use was 53% (2024) to 88% (2025) [25]. A survey focused specifically on researchers reported 76% use AI [26]. These numbers suggest that adoption is often a question of timing rather than inevitability. Together, the skills gap and the rapid emergence of new technologies underscore the need for accessible ways to explore the intersection of computational biology with other disciplines, fostering opportunities to apply computational methods in biomedical research through skill development or collaboration. This broader need for “continuing computational education” goes beyond mastering specific skills and includes maintaining an awareness of the developments in computational biology and opportunities for its application to other disciplines.

Other fields, such as medicine, offer models for structured continuing education through mechanisms like mandatory Continuing Medical Education (CME) requirements. In contrast, continuing academic education requirements are typically optional, leading to wide variability in computational expertise. There is currently no consensus on the most effective mechanisms of continuing education in computational biology for the biomedical research workforce. At the Scripps Research Institute, an independent academic biomedical research institute that also supports a graduate program, we created the Computational Biology and Bioinformatics (CBB) affinity group, a trainee-led community for the discussion of computational biology/bioinformatics with the goal of facilitating knowledge exchange. This study examines how the CBB group at Scripps Research serves as a model for addressing the above challenges, exploring CBB’s role in fostering computational literacy, promoting tool adoption, and bridging training gaps in a multidisciplinary setting.

## Results

### Assessing computational biology activity at an academic biomedical research institute

Despite the pervasive implementation of computational methods in the life sciences, quantifying the degree of computational research activity remains challenging. To gain a better understanding of computational research activity at Scripps Research, we examined official and empirical data from independent sources. As of January 2025, the institute’s website lists 24 (16%) of its 153 faculty as performing research that “contains Computational Biology or Computational Biology/Bioinformatics,” and 30 faculty members at the rank of Assistant Professor or above are affiliated with the Integrative Department of Structural and Computational Biology. However, most of these faculty also perform research in other areas. Additionally, many laboratories at Scripps rely heavily on computational methods, utilizing advanced analyses in proteomics, genomics, neuroscience, structural biology, virology, etc., without being classified as having a focus on Computational Biology. Therefore, we sought a more empirical way of assessing computational research activity at the institute.

**Fig 1:**
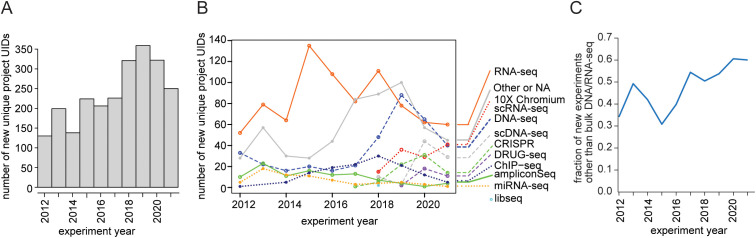
Trends in the usage of the Genomics Core. **(A)** Overall increase in unique experiments performed in the Genomics Core between 2012 and 2021. **(B, C)** Diversification in experiment types performed in the Genomics Core between 2012 and 2021. UID, unique identifier.

We identified next-generation sequencing-assisted technologies as a major area requiring computational analysis. In addition to sequencing services, the Scripps Research Genomics Core offers services including genomic and transcriptomic library preparation. We examined usage of the Genomics Core over a 10 recent year period using database records. In a dataset spanning the years 2012–2021, an increase in the number of unique experiments performed in the Core was observed: the annual number almost tripled between 2012 and 2019 (Fig 1A).

Analyzing the types of experiments performed in the core, bulk RNA-sequencing (RNA-seq) dominated as the most frequent library preparation type in most years (Fig 1B and S1 Table). However, there was a clear change in the overall composition of experiment types over time: the relative share of bulk RNA-seq decreased from a peak of 60% in 2015 to 24% in 2021, and new techniques such as 10× Chromium single-cell RNA-sequencing (scRNA-seq), CRISPR experiments, and high-throughput screening RNA-seq (such as Digital RNA with pertUrbation of Genes-seq [27]) were introduced (Fig 1B and S1 Table). The drop in the bulk RNA-seq share is only partially explained by the rise in scRNA-seq to 16.4% in 2021; the total diversity of experiment types increased significantly. Accordingly, the fraction of new experiments other than bulk RNA/DNA-seq rose from 34% in 2012 to 60% in 2021 (Fig 1C). To investigate a potential shift of bulk RNA/DNA-seq preparation from the Core to the individual research groups, we estimated the number of user-prepared libraries submitted to the Core for sequencing over time and their percentage of total core experiments for which these data were available (S1 Fig). Whereas the fraction of non-bulk-RNA/DNA-seq experiments nearly doubled between 2015 and 2020, the fraction of user-prepared libraries increased only slightly (from 45% to 52%) over the same 5-year period, suggesting user-prepared libraries are not responsible for most of this effect (S1 Fig). Overall, these data suggest that the number of unique experiments as well as the diversity of experiment types in the Genomics Core increased from 2012 to 2021, reflecting an institutional growth of activity in this research area.

We next examined aggregated usage statistics of the Scripps Research High-Performance Computing Cluster (HPC), a shared resource freely available to all institute laboratories on request. There was a slight increase in the total number of unique laboratories utilizing the HPC from 2017 to 2020 (Table 1). Notably, these numbers suggest that the majority of Scripps ~150 laboratories utilized HPC at least once during the years examined (Table 1). We observed no significant increase in the number of central processing unit (CPU) days per year; however, given improvements in software and hardware performance and utilization of additional computing resources, this parameter may not appropriately capture overall computing activity. Interestingly, laboratories utilizing the HPC belong to all Scripps Research departments, including Chemistry, The Scripps Research Translational Institute, and Molecular Medicine (now part of the Department of Molecular and Cell Biology [MCB]) (Table 2). Molecular Medicine/MCB was the top user by CPU days during the period examined, despite having fewer total research groups than the Department of Integrative and Structural Biology (ISCB) and fewer groups using HPC than ISCB, highlighting that computational biology is not confined to computational departments (Table 2 and S2 Fig). Consistent with this, the variance in total CPU usage within departments was greater than the variance between departments (S2 Fig). Multiple departments were also represented among labs whose usage increased the most year-to-year (S2 and S3 Tables). Importantly, some research groups at Scripps utilize private computational resources such as group-only clusters or cloud computing, and usage data on those resources was not available for analysis. Therefore, trends in total computational usage might differ from those of HPC usage. Moreover, changes in departmental structure and affiliations reduce the accuracy of the analysis of department-specific usage. Overall, these data reveal an active user base of genomics and computing resources at Scripps Research, spanning multiple departments.

**Table 1:** Quantification of unique research groups using HPC at Scripps between 2017 and 2020.

Year	Number of unique labs using HPC: Number (approx. % of total laboratories)
2017	83 (54%)
2018	82 (54%)
2019	85 (56%)
2020	92 (60%)

The percentage of total laboratories is an estimation due to investigators joining and leaving the institute each year, leading to fluctuations in the number of HPC-using as well as total number of laboratories.

HPC, high-performance computing cluster.

**Table 2:** Top departmental users of Scripps HPC 2017–20.

Department	Total CPU days 2017–20/ thousands	CPU days 2020/ thousands
Molecular Medicine (currently Molecular and Cell Biology)	415.5	89.4
Integrative Structural and Computational Biology	412	96.6
Chemistry	267	82.2
Immunology and Microbiology	13	0.1
Neuroscience	2.6	1.2

These totals include only labs for which data from both 2017 and 2020 were available to eliminate the confounding effect of investigators leaving Scripps within this period.

CPU, central processing unit; HPC, high-performance computing cluster.

### Supporting computational research and education at a biomedical research institute

Scripps Research offers data analysis services through its Center for Computational Biology and Bioinformatics (CCBB), a core facility. While the analysis services offered are diverse (S4 Table), the ones best utilized are predominantly genomic analyses. Although a database of specific CCBB service usage is not maintained, the approximate usage of CCBB services between April 2022 and Jan 2023 was reported to comprise mainly bulk and scRNA-seq analyses, with the remainder spread across other projects (S5 Table). Noted was also an increase in the number of scRNA-seq analyses conducted, a trend supported by more recent numbers (S6 Table). Thus, analyses for the most common data types generated in the Genomics Core are supported through the CCBB core.

**Fig 2:**
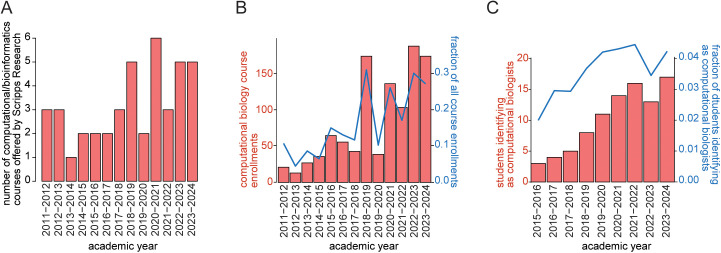
Scripps Research Skaggs Graduate School of Chemical and Biological Sciences computational/bioinformatics course offerings, enrollment, and research focus per year. **(A)** Total number of unique computational biology courses offered per year between 2011 and 2024. Full list given in S7 Table. **(B)** Total enrollment in computational biology courses per year and as a fraction of all course enrollments that year between 2011 and 2024. **(C)** Total number of students self-identifying as computational biology researchers and as a fraction of all students per year between 2015 and 2024.

The Skaggs Graduate School of Chemical and Biological Sciences at Scripps Research has consistently offered courses focused on teaching basic computational and bioinformatics skills for the past decade (Fig 2A and S7 Table). Courses range from basic introductions to coding and statistical testing in R to more advanced topics of genetics and genomics, and application of advanced statistical models. However, the number and content of these courses are limited to certain areas of computational biology, such as statistics (Fig 2A and S7 Table). To supplement these courses, the graduate studies office has supported students pursuing external courses, yet the number of students taking advantage of this benefit is very low (estimated at <5 per year). While the number of computational biology-focused courses has grown slightly, the number of students engaged in these courses has grown significantly, nearly tripling between 2011 and 2024 (Fig 2B). The total number of active students self-identifying with a computational biology research focus, which is done upon enrollment in the graduate school, showed steady increases for the 5 years since this focus became available in 2015. However, since this is also the average amount of time to graduate, this data suggests a relatively stable rate of incoming computational biology-focused students with only a minor increase (Fig 2C). Thus, it appears that the increasing student interest in learning computational biology at Scripps Research is representative of an increasing interest from scientists that do not consider themselves as primarily computational biologists, reflective of the broader trends described earlier.

Thus, institutional support for common data generation, analysis, and computational skills training is available. However, given the breadth of departments engaged in computational research and the increasing diversity of experiments performed at the Genomics Core, many scientists still must rely on asking colleagues and self-teaching to analyze their data.

### Enhancing computational research through an interest network

Our data show the scale of computational work at Scripps exceeds the formal learning opportunities present. To address this gap as well as provide a platform for information exchange, collaboration, and interdepartmental networking, we initiated the CBB interest network. The network is run by early-career scientist volunteers and aims to disseminate knowledge related to computational biology, lower the barriers to computational research, and connect scientists across disciplines and departments. To achieve these goals, the CBB has performed the following actions.

First, an active directory of members interested in computational biology at all levels of seniority has been compiled to ease collaboration and maintain a census of the computational community at Scripps Research and guests from neighboring institutions. Approximately 300 individuals have joined the CBB mailing list, of which 136 registered in the member directory. Second, a regular seminar series was launched featuring a broad range of topics connected by some degree of utilization or development of computational methods. To provide opportunities for early-career scientists, speakers from all levels of seniority have been invited, and self-nominations were welcomed. To date, 34 seminars featuring speakers from over 10 institutions have been held (Table 3). Third, seasonal workshops led by volunteers have been organized to introduce participants to specific topics of interest, such as structured query language (SQL), basics of Python grammar, etc. Fourth, coding sessions featuring code analysis, live presentation, troubleshooting, software package discussion, etc., were held intermittently. These activities were organized by a volunteer leadership group comprising early-career scientists (leadership group has fluctuated between 2 and 5 members) who meet approximately monthly; agenda and minutes were recorded, and a cloud-hosted spreadsheet of activities was maintained.

**Table 3:** Overview of Computational Biology and Bioinformatics (CBB) activities.

Activity	Number held since Feb 2020	Topics covered	Goals
Seminars	34	Summary in Fig 3.	Disseminate knowledge in computational biology. Share research updates. Facilitate collaborations. Offer presentation opportunities for early-career researchers.
Coding sessions	13	Object-oriented programming in R, lm(), and glm() modeling, CBB Github repo, Developing a Python package, Basic GNU parallel and Python multiprocessing, Automating the CBB Github repo, Standardization of visualizing scientifically accurate RNA-seq, CodeWave: AI/ML working group; Deep learning and its applications to research	Exchange programming expertise. Keep up to date with the latest software packages, statistical methods relevant to biomedical research.
Workshops	2	General code architecture, SQL (3 classes)	Provide introductory training not covered by formal courses at Scripps.

Official events organized under the CBB umbrella.

GNU, Gnu’s Not Unix; SQL, structured query language.

**Fig 3:**
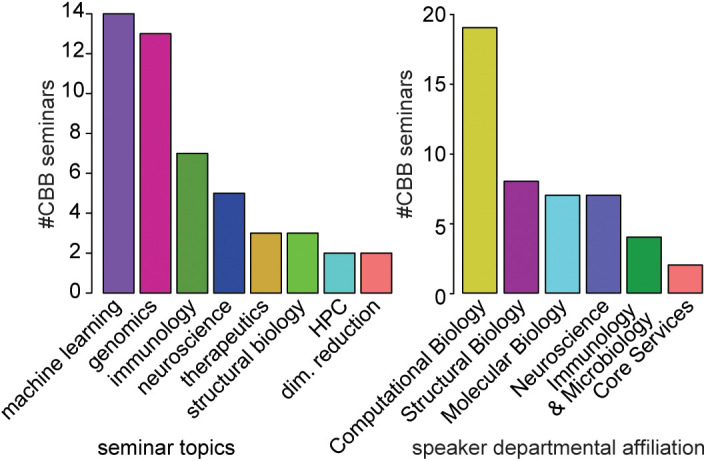
Topics and primary disciplines of Computational Biology and Bioinformatics seminars. For speakers with unknown department affiliation, department affiliation of the lab principal investigator was used. Department names were grouped by categories of research, e.g., neurology and neuroscience grouped under Neuroscience. Scientific topic for non-Scripps speakers with unknown department affiliation was assigned based on their lab website landing page as best estimate of the broad research category. Only keywords with at least 2 independent seminars were included in the frequency analysis. There were 5 singleton keywords, and 1–3 keywords were used per seminar in the seminar topics analysis.

The seminar series has operated continuously since its initiation in 2019. We examined the seminar topics to understand the range of fields represented in the series. Analyzing the top 3 keywords representing topics of interest per seminar, the most frequent keywords were genomics, machine learning, and immunology (Fig 3). However, structural biology, neuroscience, and translational science (“therapeutics”) were well represented, indicating that the seminar series transcends traditional disciplinary boundaries (Fig 3). The speakers represent 10 institutions, 6 departments at Scripps Research, and span multiple levels of seniority from graduate student to faculty (S8 Table).

**Fig 4:**
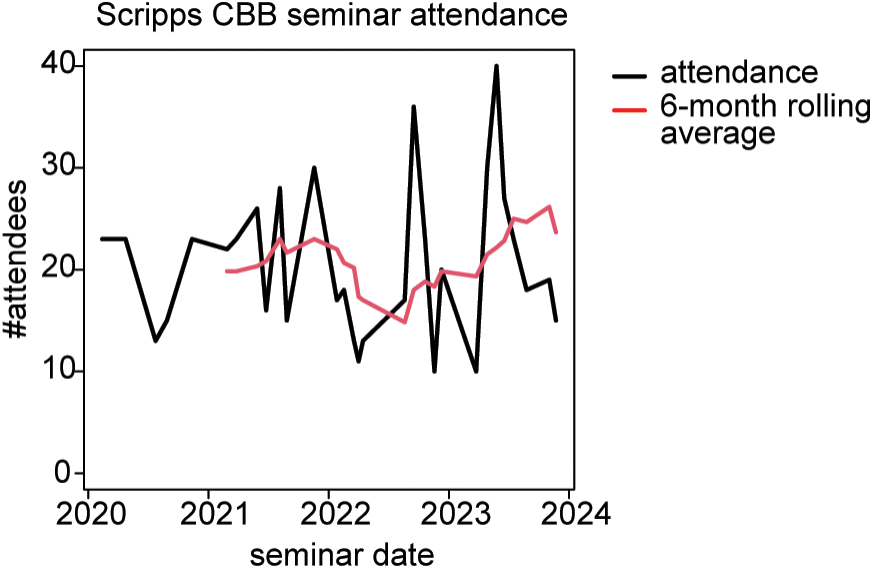
Computational Biology and Bioinformatics seminar attendance. Attendance at virtual seminars was measured as the number of unique users joining each virtual meeting.

The seminars were initially held in person but have continued in a virtual format since 2020. The median attendance as of the latest data cutoff (Nov 2023) is 24, with a trend towards increased attendance between 2021 and Nov 2023 (Fig 4). Therefore, the CBB seminar series has attracted consistent participation in its 3.5-year operation.

To advance the goal of connecting scientists interested in computational biology and exchanging knowledge and skills in this area, the CBB network organized additional activities (Table 3). A member directory was compiled and a Slack workspace launched to enable efficient contact between interactive coding sessions (held in person or virtually) and to introduce attendees to fundamental programming principles and tools directly applicable to biomedical research. Topics, among others, included object-oriented programming in R, linear models, how to develop a Python package, and advances in large language models. These coding sessions were led by CBB member volunteers, were interactive, featured opportunities for community code troubleshooting, and generally did not overlap with TSRI Graduate School courses or other formal education events on campus. This format allowed attendees to interactively learn about topics beyond what is available on campus and seek feedback on potential real-life research applications. Notably, the attendance was particularly strong during the pandemic year of 2021, highlighting the potential of interactive remote coding sessions as a means to maintain productivity when in-person research opportunities are restricted.

### Measuring the impact of the interest network

To assess the impact of CBB events on the Scripps Research community, we conducted a survey among participants in CBB initiatives. The survey was conducted over 2 months, administered electronically via email invitations to CBB members and past speakers, and collected in an anonymous manner (personally identifying information was not collected). Participants were informed that the results may be used for research purposes. All CBB members (approximately 300) received the survey invitation. A total of 28 individuals participated in the survey, an estimated participation rate of 9%.

**Fig 5:**
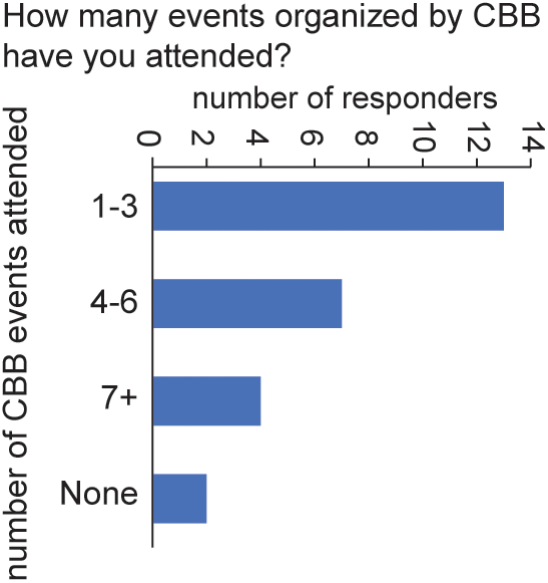
Scripps community attendance in Computational Biology and Bioinformatics (CBB)-organized events. The graph shows the distribution of the number of unique CBB events attended by each survey responder. The data were gathered in 2022 on a sample of the active Scripps community, including grad students, postdocs, full-time researchers, staff members, and PIs. The results include all 26 responses from 28 respondents who completed this question.

The first question in our survey, “How many CBB-organized events have you attended?” revealed a substantial engagement. The majority of respondents reported attending 1–3 events (13 participants), followed by 4–6 events (7 participants), and 7 or more events (4 participants). Bias in survey participation is reflected in that only two respondents indicated that they had never attended any CBB events (see Fig 5).

**Fig 6:**
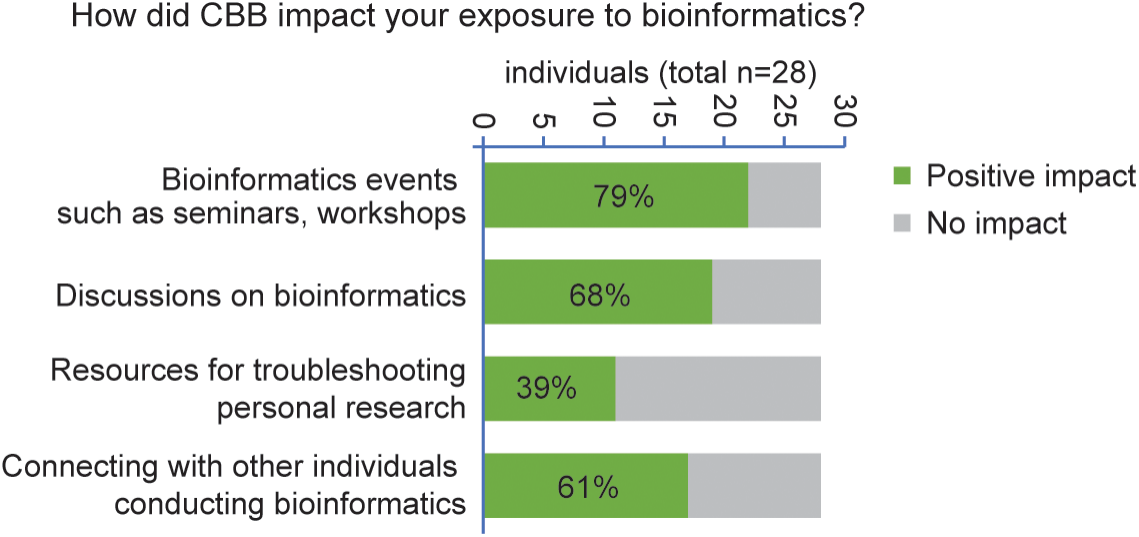
Computational Biology and Bioinformatics (CBB) impact on exposure level of the Scripps community. The graph shows the impact that CBB had on exposing members of the Scripps community to various computational-related activities. The results show a general increase in exposure to 3 out of 4 analyzed topics (Bioinformatics seminars, Discussions on bioinformatics topics, Bioinformatics network), with a total average showing an overall positive impact of CBB. The data were gathered in 2022 on a sample of the active Scripps community (*n* = 28), including grad students, postdocs, full-time researchers, staff members, and PIs. The category “positive impact” includes the following answers: “Significantly more,” “more,” “Significantly more after joining CBB,” and “More after joining CBB”; the category “No impact” includes the following responses: “Same exposure” and “Same exposure after joining CBB”.

In a follow-up question, we examined the influence of these events on the community’s exposure to bioinformatics. For most of the CBB tools and initiatives analyzed, such as Seminars and Workshops, Discussions, Resources for Troubleshooting, and Networking, the response “Positively” significantly outnumbered “Unchanged,” with an average of 17.25 compared to 10 (Fig 6). Notably, the only CBB initiative that did not have a predominantly positive impact on the community was “Resources for Troubleshooting,” with 11 respondents indicating a positive impact compared to 17 who reported no change.

**Fig 7:**
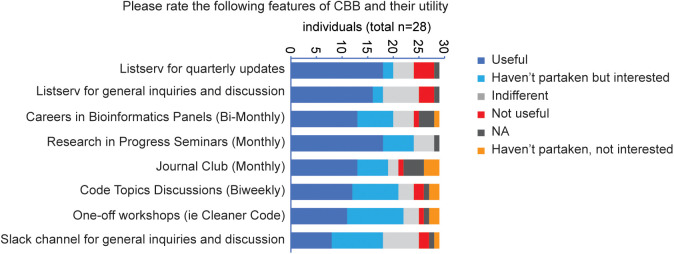
Scripps community evaluation on Computational Biology and Bioinformatics (CBB)-organized events and tools. The graph shows how the responders evaluated some of the CBB features. NA, not applicable.

In our final survey question, we asked participants to rate the usefulness of various CBB features (“Listserv for quarterly updates,” “Listserv for general inquiries and discussion,” “Careers in Bioinformatics Panel,” “Research in Progress Seminars,” “Journal Club,” “Code Topic Discussions,” “One-off Workshops,” and “Slack channel for general inquiries and discussions”) across five categories: “Useful,” “Not partaken but interested,” “Indifferent,” “Not useful,” and “Not partaken and not interested.” On average, 13.2 of 28 respondents found CBB features “Useful,” 6.2 expressing interest but being unable to participate, 4.2 indicating indifference, 1.7 finding them “Not useful,” and only 1.1 respondents who had neither participated nor expressed interest (see Fig 7). The seminar series attracted the most interest overall (Fig 7).

Although the sample of 28 respondents is unlikely to be representative of the Scripps community at large, these results demonstrate that CBB activities have had a positive impact on at least some individuals, resulting in increased exposure to information related to computational biology and connecting researchers interested in the field, with most activities deemed useful by those who participated. Therefore, despite the limited extrapolative potential of these results, the survey was conclusive in confirming the presence of a community of CBB members who benefited from participation in CBB. The results show a general positive evaluation for all the categories under examination, reflected by the highest average value for “Useful,” followed by “Haven’t partaken but interested” and “Indifferent,” indicating CBB programming has been well received. Although we were unable to directly evaluate the effect on computational literacy, these results suggest CBB has increased interdisciplinary community engagement with computational biology and access to learning resources.

## Discussion

Our findings demonstrate that the Scripps Research CBB group has effectively engaged researchers across disciplines, fostering community participation and promoting knowledge exchange in computational biology. Our analyses of the Genomics Core and the HPC cluster have shown that next-generation sequencing experiments have diversified, and computationally intensive applications are not restricted to research groups formally devoted to computational biology. These trends highlight the need for a community that shares computational expertise across this wide range of biological fields in which computational methods are now routinely applied. Quantitative metrics, including attendance trends and participant feedback, indicate a growing demand for computational training and broad approval of this trainee-led initiative. While direct assessment of the CBB’s impact on bridging specific skill gaps was not feasible—given the open-access nature of events and their diverse scientific focus—the sustained engagement and consistent demand suggest that the group addresses a critical unmet need in postgraduate computational education. Although the survey sample size (*n* = 28) is limited and likely subject to responder bias, the overwhelmingly positive feedback underscores the value of the CBB for a subset of the Scripps Research community, highlighting its role as a complementary mechanism for computational skill development. Importantly, CBB activities addressed the broader need of continuing computational education by increasing exposure to applications of computational biology to real-world research projects and creating opportunities for networking and collaboration.

Analysis of graduate course offerings and core service usage reveals that institutional support for computational research is available through recurring courses and analysis services. However, this support has inherent limitations, particularly for specialized or tailored analyses, due to the constrained variety of computational courses and selective use of custom analysis services. Given the specialized and rapidly evolving nature of computational research methods, institutional resources must prioritize certain approaches over others, limiting their ability to serve as a comprehensive solution. Additionally, reliance on institutional support services is particularly challenged in situations of reduced federal research funding, as these services are often some of the first to be cut.

We do not advocate for a compulsory curriculum like CME for academic researchers, but note that the unstructured nature of individual development often leaves low-yield areas unaddressed. Bioinformatics advances can seem irrelevant to nonexperts due to perceived low impact or high time costs of implementation, but ignoring all technological advances risks loss of innovation and efficiency. The optimal compromise is likely to depend on project details and rate of methodological development in the area. Thus, for promoting workforce-wide bioinformatics competency, approaches beyond compulsory training and completely independent study should be considered [28,29]. Leaning on communities of practice is one alternative approach that leverages benefits of flexible group/situated learning [30–33], and we developed the CBB as a mechanism to augment institutional resources and report evidence supported by data from the CBB case study. This mirrors the reports of other communities of practice similarly developed within academic [34,35] and healthcare contexts [36–39], including those with computational biology focus [40–42]. By pairing descriptions of the creation, operation, and impact of the CBB with detailed institutional support context via the HPC and CCBB cores and graduate school course offerings, we add to prior literature on communities of practice operating within the biomedical research space. We expect this added context to be particularly important for the evaluation of communities of practice, both generally, as social learning structures, and specifically, to the biomedical research space where significant institutional support already exists.

Community-directed programming led by communities of practice offers important advantages over existing institutional resources. Activities led by communities of practice naturally tailor closely to the evolving interests of the group, as this is required for their survival [43]. This fluidity overcomes the institutional rigidity of any one department, allowing for exploration of topics that may have traditionally been treated as out of scope.

Workshops hosted by larger groups such as European Bioinformatics Institute and Cold Spring Harbor Laboratories, or online, can also provide an excellent means for finding such a community, especially where the topic of interest is particularly niche or where local organizers are lacking. However, local communities have complementary advantages derived from being embedded in the same space where the research is performed. This increases direct application of knowledge (e.g., How do I access our HPC? How do I perform and analyze scRNA-seq in my institution?) and ease of engagement. Therefore, due to the limits of delocalization of biomedical research, online communities of practice and local communities of practice offer nonredundant services to their members. Further research into the interaction of local and larger/online communities of practice would help direct ways in which they can complement one another’s strengths.

After trialing multiple event types, we found that the seminar approach was particularly well received, earning the highest approval rating from survey respondents. This likely reflects the relevance of seminar topics to group members, presented in a familiar format that supports varying degrees of engagement. Some seminars provided a deeper dive into tools/technologies, and others into the utilization of such tools/technologies to particular fields. Both active and passive participation provide benefits [31]. In contrast, our one-off workshops mostly targeted newer members of the CBB; however, this could be expanded in case of sufficient enthusiasm/interest in advanced topics. Additionally, while we made some attempts to centralize information for reuse (e.g., seminar recordings as permitted), creation of crowd-sourced wikis/summaries is another approach we would suggest for accumulating advice. An additional benefit of communities of practice transcends the formal programming-related learning opportunities they provide and is tied to the social networks they facilitate. These networks play a large part in setting organizational culture and promoting a sense of belonging [44]. Together, these elements are critical to an institute’s identity and help to enhance its constituents’ performance [44]. The survey results show that the CBB’s programming provided opportunities for participants to connect with other individuals (Fig 6), likely supporting these downstream effects. Although the survey conclusively demonstrated that some members of CBB benefit from participation, the low absolute number of respondents limits our ability to extrapolate from these results and suggests an alternative survey method may be required to identify areas for future growth and improvement. Another important limitation of this study is that the direct impact of CBB on research productivity was not examined. This important question may require a prospective approach with careful methodological design to assess the impact of CBB independent of other effects, a challenge given the difficulty of a randomized controlled design in this context.

Logistically, the operation of CBB has required a core volunteer group of 2–5 early-career scientists who devoted a minimum of approximately 1 h per week in CBB-specific efforts (but exceeding 10 h per week during peak activities such as workshops or data analysis). The only other prerequisites include access to a virtual meeting platform, conference rooms, and institute mailing lists. No funding was required to establish and run the CBB group, although departmental support in the form of administrative assistance and occasional light refreshments for in-person seminars was provided. Therefore, the minimum operating requirements are low. The limiting factor to community-led programming in our experience has been community enthusiasm/interest. The level of this factor is intrinsic to each community. While we show the enthusiasm/interest in the CBB at Scripps Research has been sufficient to support seminar series, we found it was insufficient to support consistent programming for additional series, such as journal clubs or workshops. Factors influencing engagement are often beyond the control of organizers, but leadership buy-in is a modifiable factor. Anecdotally, labs that emphasize individual development, both formal and informal, tended to have greater participation. To foster broader engagement, we recommend institutional support of community-driven learning, ensuring that group activities are recognized as productivity-enhancing rather than productivity-competing. This can be aided by emphasizing alignment between professional development goals (including teaching, leadership, and research advancement) with the goals of the community of practice. Future studies should investigate how communities of practice impact computational skill level and productivity in biomedical research environments.

## Materials and methods

### Ethics statement: Human subjects

The survey was reviewed by the Institutional Review Board (IRB) of Scripps Health and Scripps Research (IRB-24-8318). The IRB confirmed the survey qualifies for exemption from full review in accordance with article 45 CFR 46.104(d) (2 ). The study complied with ethical standards in the Belmont Report. No informed consent was used as all survey responses were collected anonymously.

### Genomics core analysis

We used de-identified data on TSRI Genomics Core experiments over 10 years (2012–2021), exported from an SQL database utilized routinely for experiment tracking. The following fields were used to identify experiments: project_uid, creation_date, sample_type. The project_uid was used as a unique identifier of experiments (typically, each discrete experiment would be assigned a unique project_uid). The sample_type column was used to identify experiment types and manually converted into a new sample_type_simplified column to group together experiments of the same type (for example, polyA+ and ribosome depletion RNA-seq library preparation protocols would be grouped under RNA-seq). Experiments not fitting in any major categories were assigned “Other_or_NA” in the sample_type_simplified column. The assigned_to column was used to determine which libraries were prepared by the core staff vs the submitting investigator (user-prep). R versions 4.2 and 4.5 were used for analysis and plotting. Primary data and code are available on GitHub.

### High-performance cluster analysis

We used de-identified data on the main computing cluster at TSRI over 4 years (2017–2020). The following fields were analyzed: Year, Group, CPU days, and Number of jobs. The departmental affiliation of each research group was determined using data from 2022 or the closest available year to the dates analyzed. The Department of Molecular Medicine was merged with the Department of MCB, with most MM faculty becoming members of MCB in 2023; therefore, MM and MCB are combined as a single department for the purpose of this analysis. R versions 4.2 and 4.5 were used for analysis and plotting. GraphPad Prism version 10 was used for bar graph/dot plot generation.

### Course offering and enrollment analysis

We compiled Scripps Research Skaggs Graduate School of Chemical and Biological Sciences course offerings related to computational/bioinformatics topics within the period between 2011 and 2024 (S7 Table). We used de-identified course enrollment information to calculate the total number of enrollments in these courses and their fraction of total course enrollments that year. Primary data and code available on GitHub. R version 4.2 was used for analysis and plotting.

### Student research focus selection

We used de-identified student research interest information from the Scripps Research Skaggs Graduate School of Chemical and Biological Sciences over the period between 2011 and 2024 to determine the total number of students identifying as computational biologists in this period and the fraction of total students this represented on a per-year basis. Student research interest information was collected by the graduate school, with each student self-selecting a primary designation upon enrollment in the graduate program. Primary data and code available on GitHub. R version 4.2 was used for analysis and plotting.

### Seminar topic analysis

Seminar information, including location, host, attendance, speaker, home lab, seminar title, and date delivered, was recorded beginning in February 2020. If the presenter was a principal investigator (PI), their department affiliation was used, and if not, their PI’s departmental affiliation was used as the department of speaker. Speakers with unknown departmental affiliations were marked as NA and excluded from the affiliation analysis. Speakers from core services units were assigned an independent department “Core Services.” To harmonize diverse department names, categories of research, e.g., neurology => neuroscience, were supplied to “Field Categories.” To designate scientific topic for non-Scripps speakers with unknown Department affiliation, their lab website landing page was used to best estimate the broad research category. Only keywords with at least 2 independent seminars were included in the frequency analysis. There were 5 singleton keywords. This yielded a list including Genomics, Machine Learning, Immunology, Neuroscience, Therapeutics, Structural Biology, High Throughput Computing, Dimensionality Reduction, and Proteomics. R version 4.2 was used for analysis and plotting.

### Survey data collection

The survey comprised several questions designed to gauge the extent of community involvement in CBB events and the perceived impact of these initiatives. The primary questions focused on the frequency of attendance at CBB-organized events, the influence of these events on exposure to bioinformatics, and the usefulness of various CBB features.

The survey was distributed on three occasions over a 2-month period to mailing lists that included CBB members and early-career scientists (graduate students, postdocs). Utilizing Google Forms, survey data were systematically collected. To uphold respondent anonymity, no personal identifiers were collected. Respondents were explicitly instructed not to submit their responses more than once to prevent duplication. Complete survey data is available as S1 File.

### Survey data analysis

Quantitative analysis of survey responses was performed to discern trends and patterns within the collected data using Python 3 and, Pandas library. Descriptive statistics were employed to summarize participant demographics, attendance rates at CBB events, and perceptions regarding the impact and utility of CBB initiatives. The frequency distribution of respondents’ attendance at CBB events was analyzed to understand the level of community engagement. The number of events attended by each participant was categorized, and the distribution was visualized using graphical representation. Responses regarding the influence of CBB events on community exposure to bioinformatics (Fig 6) and rating of CBB features (Fig 7) were analyzed by grouping the responses from a question per each category expressed as bar in the plot. The frequency of positive, neutral, and negative perceptions was tallied for each CBB initiative or feature, and comparative analysis was conducted to identify trends and areas for improvement. Participants’ ratings of various CBB features were analyzed to assess their perceived usefulness. Responses were categorized into “Positively” or “Unchanged” for the services, or distinct ratings, such as “Useful,” “Interested but unable to participate,” “Indifferent,” “Not useful,” and “Not interested,” for the features, and the distribution across these categories was examined. Microsoft Excel was used for survey results plotting.

## Data availability

Code used for analysis and plotting, source data and the list of CBB seminars are available at the following GitHub repository: SuLab/TSRI-CBB. Additional source and processed data are available as Supplementary Information.

## Supporting information

S1 Fig Contribution of user-prepared next-generation sequencing libraries to overall sample pool in the Genomics Core. A subset of Core usage data with information on whether the library was prepared by Core staff or outside the Core (user-prep) was analyzed by year of submission. Red line shows the percentage of submissions attributed to user-prepped libraries; gray and blue bars represent the number of total and user-prepared submissions, respectively.
(PDF)

S2 Fig Utilization of the High-Performance Computing cluster in CPU days by department. Chem, Chemistry; CPU, central processing unit; IMM, Immunology and Microbiology; ISCB, Integrative and Structural Biology; MCB, Molecular and Cell Biology; MM, Molecular Medicine; Neuro, Neuroscience. The departmental affiliations of research groups were determined using 2022 data or closest available data relative to the last year of data collection (2020).
(PDF)

S1 Table Number of experiments by category performed in the Genomics Core. (PDF)

S2 Table Department affiliations of research groups with largest usage increase and decrease from 2017 to 2020. There is no obvious bias in the department affiliations of labs which increased vs decreased their HPC usage. There is no statistical enrichment of ISCB labs among HPC users who increased vs. decreased usage. HPC, High-Performance Computing cluster; ISCB, Integrative Structural and Computational Biology; MM, Molecular Medicine; SRTI, Scripps Research Translational Institute.
(PDF)

S3 Table Departmental affiliations of groups which increased and decreased their HPC usage as measured by CPU hours. ISCB, Integrative Structural and Computational Biology; MM, Molecular Medicine.
(PDF)

S4 Table Services offered by the CCBB. Scripps Research California core analysis services are offered as of 1/11/2024. The most up-to-date offerings can be found on the institute’s web pages. ATAC, assay for transposase-accessible chromatin; CCBB, Center for Computational Biology and Bioinformatics; CNV, copy number variant; GEO, Gene Expression Omnibus; NCBI, National Center for Biotechnology Information; SNP, single-nucleotide polymorphism; SRA, Sequence Read Archive; UMI, unique molecular identifier.
(PDF)

S5 Table Approximate CCBB service usage between April 2022 and January 2023. (PDF)

S6 Table CCBB analyses requested in the academic years from 2022 to 2025. (PDF)

S7 Table Courses offered by Scripps Research (CA and FL) covering computational/bioinformatics topics between 2011 and 2024. (PDF)

S8 Table Details on CBB Monthly Seminar speakers. (PDF)

S1 File Spreadsheet of CBB survey results. (XLSX)
