# Supplementary material for: Catalyzing computational biology research at an academic institute through an interest network
Source: PLoS Comput Biol. 2025 Sep 10;21(9):e1013453. doi: 10.1371/journal.pcbi.1013453 (PMC12422415; doi:10.1371/journal.pcbi.1013453)
Supplement: S1 Fig — A subset of Core usage data with information on whether the library was prepared by Core staff or outside the Core (user-prep) was analyzed by year of submission. Red line shows the percentage of submissions attributed to user-prepped libraries; gray and blue bars represent the number of total and user-prepared submissions, respectively. (PDF) [file pcbi.1013453.s001.pdf]

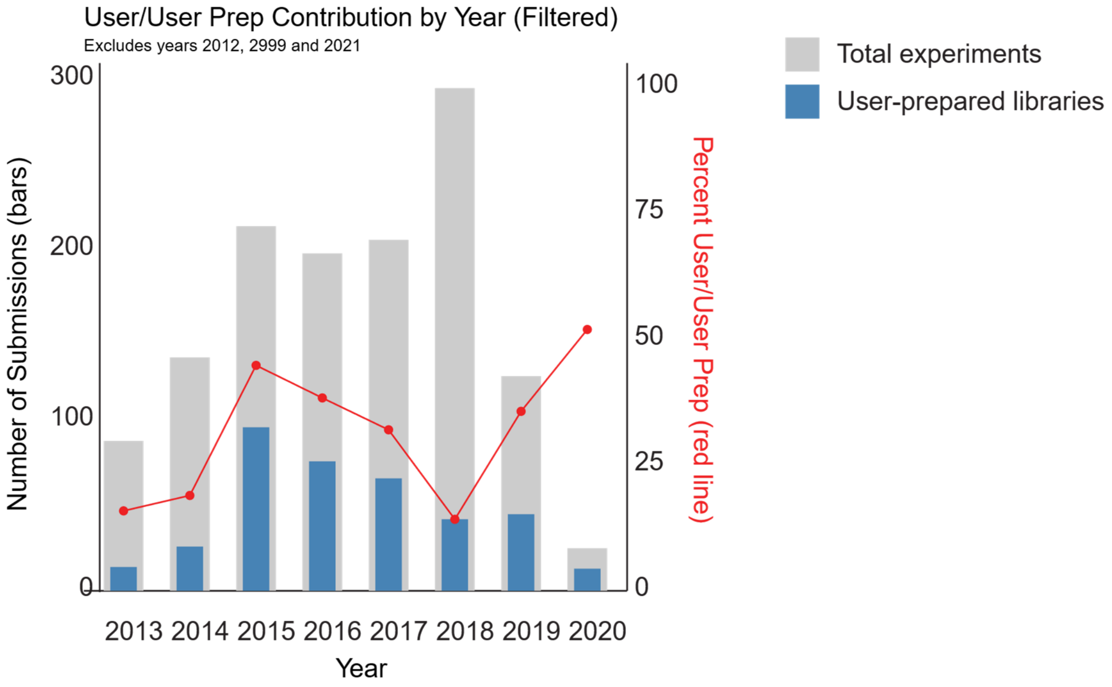

S1 Fig. Contribution of user-prepared next-generation sequencing libraries to overall sample pool in the Genomics Core. A subset of Core usage data with information on whether the library was prepared by Core staff or outside the Core (user-prep) was analyzed by year of submission. Red line shows the percentage of submissions attributed to user-prepped libraries; grey and blue bars represent the number of total and user-prepared submissions, respectively.
