# Supplementary material for: Catalyzing computational biology research at an academic institute through an interest network
Source: PLoS Comput Biol. 2025 Sep 10;21(9):e1013453. doi: 10.1371/journal.pcbi.1013453 (PMC12422415; doi:10.1371/journal.pcbi.1013453)
Supplement: S2 Fig — Chem, Chemistry; CPU, central processing unit; IMM, Immunology and Microbiology; ISCB, Integrative and Structural Biology; MCB, Molecular and Cell Biology; MM, Molecular Medicine; Neuro, Neuroscience. The departmental affiliations of research groups were determined using 2022 data or closest available data relative to the last year of data collection (2020). (PDF) [file pcbi.1013453.s002.pdf]

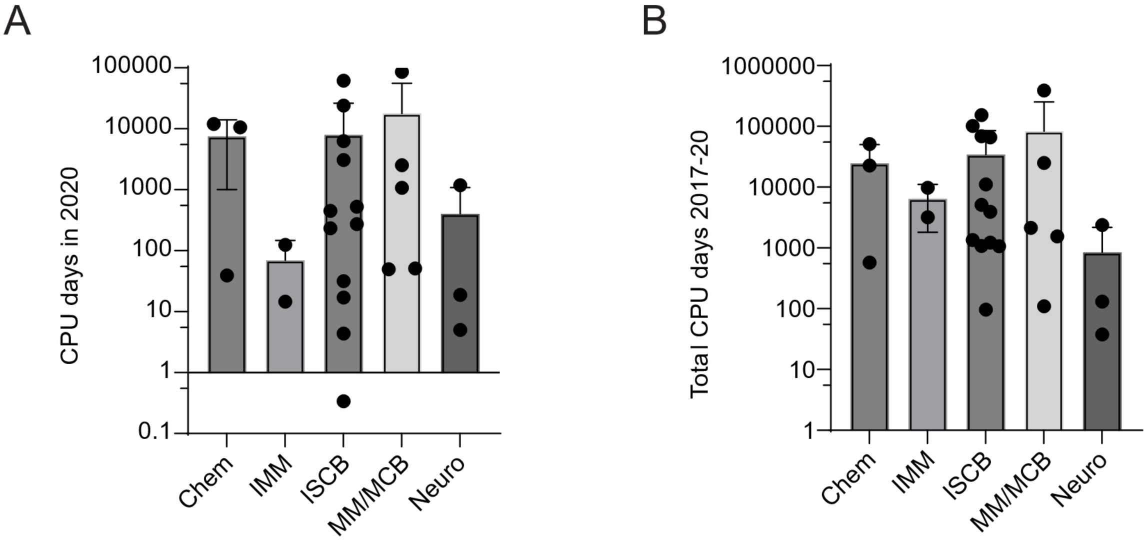

S2 Fig. Utilization of the High-Performance Computing cluster in CPU days by department. Chem, Chemistry; CPU, central processing unit; IMM, Immunology and Microbiology; ISCB, Integrative and Structural Biology; MM, Molecular Medicine; MCB, Molecular and Cell Biology; Neuro, Neuroscience. The departmental affiliations of research groups were determined using 2022 data or closest available date to the last year of data collection (2020).
