# Supplementary material for: Catalyzing computational biology research at an academic institute through an interest network
Source: PLoS Comput Biol. 2025 Sep 10;21(9):e1013453. doi: 10.1371/journal.pcbi.1013453 (PMC12422415; doi:10.1371/journal.pcbi.1013453)
Supplement: S1 Table — (PDF) [file pcbi.1013453.s003.pdf]

S1 Table. Number of experiments by category performed in the Genomics Core.

	Experiment type	2012	2013	2014	2015	2016	2017	2018	2019	2020	2021
1	RNA-seq	52	79	64	135	108	82	111	78	62	60
2	DNA-seq	33	22	16	20	16	21	48	88	65	40
3	Other or NA	28	57	30	28	44	84	89	100	57	45
4	ChIP-seq	10	23	11	16	12	13	7	4	1	4
5	miRNA-seq	5	18	12	11	7	3	4	5	3	1
6	Amplicon-seq	1	0	5	14	19	22	30	21	12	5
7	CRISPR	0	0	0	0	0	1	5	22	31	14
8	10X Chromium RNA-seq	0	0	0	0	0	0	15	36	29	41
9	Lib-seq	0	0	0	0	0	0	10	3	44	29
10	scDNA-seq	0	0	0	0	0	0	2	0	0	0
11	DRUG-seq	0	0	0	0	0	0	0	2	18	11
