# Supplementary material for: Catalyzing computational biology research at an academic institute through an interest network
Source: PLoS Comput Biol. 2025 Sep 10;21(9):e1013453. doi: 10.1371/journal.pcbi.1013453 (PMC12422415; doi:10.1371/journal.pcbi.1013453)
Supplement: S2 Table — There is no obvious bias in the department affiliations of labs which increased vs decreased their HPC usage. There is no statistical enrichment of ISCB labs among HPC users who increased vs. decreased usage. HPC, High-Performance Computing cluster; ISCB, Integrative Structural and Computational Biology; MM, Molecular Medicine; SRTI, Scripps Research Translational Institute. (PDF) [file pcbi.1013453.s004.pdf]

S2 Table. Department affiliations of research groups with largest usage increase and decrease from 2017-2020. There is no obvious bias in the department affiliations of labs which increased vs decreased their HPC usage. There is no statistical enrichment of ISCB labs among HPC users who increased vs decreased usage. ISCB, Integrative Structural and Computational Biology; MM, Molecular Medicine; SRTI, Scripps Research Translational Institute; HPC, High Performance Computing cluster.

	**Usage increase: fold**	**Usage increase: hours**	**Department**
Lab 1	4X	60k	ISCB
Lab 2	1.2X	14k	Chemistry
Lab 3	2X	12k	ISCB
Lab 4	6X	8.7k	MM
Lab 5	3X	1.7k	SRTI
Lab 6	100% drop	2.3k	Chemistry
Lab 7	100% drop	7.5k	ISCB
