# Supplementary material for: Catalyzing computational biology research at an academic institute through an interest network
Source: PLoS Comput Biol. 2025 Sep 10;21(9):e1013453. doi: 10.1371/journal.pcbi.1013453 (PMC12422415; doi:10.1371/journal.pcbi.1013453)
Supplement: S3 Table — ISCB, Integrative Structural and Computational Biology; MM, Molecular Medicine. (PDF) [file pcbi.1013453.s005.pdf]

S3 Table. Departmental affiliations of groups which increased and decreased their HPC usage as measured by CPU days. ISCB, Integrative Structural and Computational Biology; MM, Molecular Medicine.

	**Number of labs increased usage**	**Number of labs decreased usage**
ISCB (excluding emeriti)	4	7
MM	2	2
