# Supplementary material for: Catalyzing computational biology research at an academic institute through an interest network
Source: PLoS Comput Biol. 2025 Sep 10;21(9):e1013453. doi: 10.1371/journal.pcbi.1013453 (PMC12422415; doi:10.1371/journal.pcbi.1013453)
Supplement: S4 Table — Scripps Research California core analysis services are offered as of 1/11/2024. The most up-to-date offerings can be found on the institute’s web pages. ATAC, assay for transposase-accessible chromatin; CCBB, Center for Computational Biology and Bioinformatics; CNV, copy number variant; GEO, Gene Expression Omnibus; NCBI, National Center for Biotechnology Information; SNP, single-nucleotide polymorphism; SRA, Sequence Read Archive; UMI, unique molecular identifier. (PDF) [file pcbi.1013453.s006.pdf]

S4 Table. Services offered by the CCBB.

Scripps Research California core analysis services offered as of 1/11/2024. The most up to date offerings can be found on the institute's web pages. ATAC, assay for transposase-accessible chromatin; CCBB, Center for Computational Biology and Bioinformatics; CNV, copy number variant; GEO, Gene Expression Omnibus; NCBI, National Center for Biotechnology Information; SNP, single-nucleotide polymorphism; SRA, Sequence Read Archive; UMI, unique molecular identifier.

**Analysis Category**	**Analyses**
Transcriptomics	RNA-seq, UMI-based RNA-seq, High throughput screening RNA-seq ("DRUG-seq"), smallRNA-Seq (miRNA-seq)
Single-cell analytics	Gene expression, Immune profiling (VDJ, VDJ+Gene expression), Epigenome profiling (ATAC, Multiome ATAC + Gene expression), SNP-based demultiplexing of multiplexed data
Spatial genomics	Visium Spatial transcriptomics (10X Genomics), GeoMx Digital Spatial Profiling (nanoString)
Genomics	Whole Exome, Whole Genome sequencing data analyses, SNPs, indels, CNVs
Epigenomics	ChIP-Seq/eCLIP-Seq/CUT&RUN/RIP-Seq, ATAC-seq
Metagenomics	16S rRNA-Seq
Custom analyses	Sequencing data analyses for specific lab projects that use standard tools, software packages and/or custom code/script developed by CCBB.
Public datasets	Analyses on public dataset available at NCBI's GEO database repository
Functional analyses	Advaita's iPathwayGuide, Gene Set Enrichment Analysis (GSEA)
Consultations	We provide consultations on experimental design, overview of analyses and costs involved. We can take on both short-term (typically less than a month and about 10 hours of CCBB effort) and long-term collaborative projects involving bioinformatic support.
GEO/SRA uploads	We can submit sequencing data, related sample metadata and the results of analyses to NCBI repositories like GEO and SRA for a small service fee.
