# Supplementary material for: Catalyzing computational biology research at an academic institute through an interest network
Source: PLoS Comput Biol. 2025 Sep 10;21(9):e1013453. doi: 10.1371/journal.pcbi.1013453 (PMC12422415; doi:10.1371/journal.pcbi.1013453)
Supplement: S5 Table — (PDF) [file pcbi.1013453.s007.pdf]

S5 Table. Approximate CCBB service usage between April 2022 and Jan 2023.

Service	Approximate usage
RNA-seq (and variations)	40
Single cell 10X datasets (gene expression, VDJ, multi) analyses	20
Other projects (miRNA-seq, ChIP-seq, DNA-seq/misc. custom, microbiome, extensions to standard pipeline analyses)	60
Short-term and long-term collaborative work	4
Distribution of FASTQs/raw BCL data	75
GEO submissions	3
GSEA/Advaita pathway analyses	20
