# Supplementary material for: Catalyzing computational biology research at an academic institute through an interest network
Source: PLoS Comput Biol. 2025 Sep 10;21(9):e1013453. doi: 10.1371/journal.pcbi.1013453 (PMC12422415; doi:10.1371/journal.pcbi.1013453)
Supplement: S6 Table — (PDF) [file pcbi.1013453.s008.pdf]

S6 Table. CCBB analyses requested in the academic years from 2022 to 2025.

Data Analysis Service	2022-2023	2023-2024	2024-2025
Single cell RNA-seq	20	18	41
Bulk RNA-seq	40	18	21
Custom/other	60	24	48
