# Supplementary material for: Catalyzing computational biology research at an academic institute through an interest network
Source: PLoS Comput Biol. 2025 Sep 10;21(9):e1013453. doi: 10.1371/journal.pcbi.1013453 (PMC12422415; doi:10.1371/journal.pcbi.1013453)
Supplement: S7 Table — (PDF) [file pcbi.1013453.s009.pdf]

S7 Table. Graduate courses offered by Scripps Research (CA and FL) covering computational/bioinformatic topics between 2011-2024.

Academic Year	Number of computational/bioinformatic courses offered	Course titles	Total number of courses offered
2011-2012	3	Basic Biostatistics, Human Genetics and Genomics, Applied Bioinformatics & Computational Biology	23
2012-2013	3	Introduction to Biostatistics, Human Genetics and Genomics, Applied Bioinformatics & Computational Biology	29
2013-2014	1	Introduction to Biostatistics	17
2014-2015	2	Introduction to Biostatistics, Genetics and Genomics	29
2015-2016	2	Introduction to Biostatistics, Applied Bioinformatics and Computational Biology	22
2016-2017	2	Introduction to Biostatistics, Genetics & Genomics	28
2017-2018	3	Quantitative Data Analysis Bootcamp, Introduction to R, Introduction to Biostatistics	24
2018-2019	5	Quantitative Data Analysis Boot Camp, Fundamentals of Scientific Computing, Applied Bioinformatics and Computational Biology, Introduction to Biostatistics, Genetics and Genomics	33
2019-2020	2	Quantitative Data Analysis Boot Camp, Introduction to Biostatistics	24
2020-2021	6	Quantitative Data Analysis Boot Camp, Fundamentals of Scientific Computing, Applied Bioinformatics and Computational Biology, Advanced Methods in Statistical Analysis, Introduction to Biostatistics, Genetics and Genomics	30
2021-2022	3	Quantitative Data Analysis Boot Camp, Introduction to Biostatistics, Computational and Analytical Tools for Chemists	26
2022-2023	5	Quantitative Data Analysis Boot Camp, Fundamentals of Scientific Computing, Applied Bioinformatics and Computational Biology, Introduction to Biostatistics, Advanced Methods in Statistical Analysis	31
2023-2024	5	Advanced Methods in Statistical Analysis, Introduction to Data Science, Introduction to Biostatistics, Advanced Data Science, Computational and Analytical Tools for Chemists	32
