# Supplementary material for: Catalyzing computational biology research at an academic institute through an interest network
Source: PLoS Comput Biol. 2025 Sep 10;21(9):e1013453. doi: 10.1371/journal.pcbi.1013453 (PMC12422415; doi:10.1371/journal.pcbi.1013453)
Supplement: S8 Table — (PDF) [file pcbi.1013453.s010.pdf]

S8 Table. Details on CBB Monthly Seminar speakers.

Career stage	Number of speakers
PhD student	5
Postdoc	14
Staff scientist	2
Faculty	8
Industry scientist	6
Core facility scientist	2
